# Impact of cuticle photoluminescence on the color morphism of a male damselfly *Ischnura senegalensis* (Rambur, 1842)

**DOI:** 10.1038/srep38051

**Published:** 2016-12-14

**Authors:** Chin-Jung Chuang, Cheng-Der Liu, Ranjit A. Patil, Chi-Chung Wu, Yao-Chih Chang, Chih-Wen Peng, Ting-Kwuan Chao, Je-Wen Liou, Yung Liou, Yuan-Ron Ma

**Affiliations:** 1 Department of Opto-Electronic Engineering, National Dong Hwa University, Hualien, 97401, Taiwan; 2 Institute of Medical Sciences, Tzu Chi University, Hualien 97004, Taiwan; 3 Department of Physics, National Dong Hwa University, Hualien, 97401, Taiwan; 4 Department of Biochemistry, School of Medicine, Tzu Chi University, Hualien 97004, Taiwan; 5 Institute of Physics, Academia Sinica, Taipei, 11529, Taiwan

## Abstract

In this study the damselfly *Ischnura senegalensis* (Rambur, 1842) was first found to produce strong photoluminescence (PL) emissions from various colored-body portions, such as the eighth abdominal segment of the tail. The colors of the colored-body portions can be enhanced or modified by the PL emissions for assistance in reducing intrasexual and male harassment, and improving mature mating and conspecific identity. Therefore, the PL emissions that contribute to the color modification and coloration are involved in the cuticle evolution of the damselflies. The micro-PL confocal images verify that the PL emissions can strongly influence the surface colors of the cuticle, and demonstrate why the damselfly *Ischnura senegalensis* is called a bluetail.

The American epic science-fiction film “Avatar” (directed, written, co-produced, and co-edited by James Cameron) takes place on a lush habitable moon named Pandora, which has a variety of fluorescent species. Actually, the Earth can be likened to the fictional Pandora, because it also has a large number of fluorescent species, including plants, animals and trees^1^,^2^. One common example is the firefly; however, the fluorescence of the fireflies is categorized as a type of bioluminescence (BL) that is different from photoluminescence (PL). BL is a kind of visible-light emission generated by the chemical reactions of luciferase and oxygen^3^,^4^,^5^,^6^, while PL is a low-intensity visible-light emission excited by high-energy incident photons, such as ultraviolet (UV) light. Many fluorescent species found in nature (e.g., the damselflies examined in this study) give off PL emissions, but they are difficult to see both in the daytime and nighttime. This is because the sun light is too strong in the daytime, and there is no excited light present in the nighttime. If it is hard to see the PL, why do they (in this case damselflies) need these PL emissions. Why did they develop this capability during the course of evolution? In fact, PL emissions can be seen in the daytime, and make the color different. For example, the white paper and shirts always are added with fluorescent whitening agents, which can produce PL emissions to make them look whiter under lights. Hence, PL emissions must offer some advantage in the natural habitat of damselflies. Here we examine the PL emissions from the various colored-body portions of the male damselfly *Ischnura senegalensis* (Rambur, 1842), such as the compound eyes and the eighth abdominal segment of the tail, because the males exhibit a color mono-morphism^7^. *Ischnura senegalensis* (Rambur, 1842), also known as the common bluetail, is a common type of damselfly that is widespread in Africa, the Middle East, and throughout southern and eastern Asia. They usually inhabit clean shallow water, and are easily found in running water and ponds.

Unlike the monomorphic males, the female damselfly *Ischnura senegalensis* (Rambur, 1842) exhibit color dimorphism (namely, andromorph and gynomorph, as shown in Fig. S1(a), Supplemental Information). The andromorph helps to reduce intrasexual and male harassment and allows them to gain mature mating (as shown in Fig. S1(b), Supplemental Information)^7^. The andromorphic colors may be used as signals to improve sexual selection^8^, because they provide good visual contrast for conspecific identity in the ambient light of a varying environmental background. The capability of assuming conspecific identity provides not only a better chance for sexual selection, but also an advantage in territorial defense^9^,^10^,^11^,^12^. In this study, the PL emissions from the cuticles of various colored-body portions of the male damselfly were observed and verified in PL spectromicroscopy, and confocal fluorescence microscopy. Obviously, the intensity of the PL emissions embodies a color sign of health and strength (as shown in Fig. S2, Supplemental Information). For example, a high PL intensity suggests the good health and strength of the damselfly. Other damselflies observe this. The males will choose not to intrude on its territorial airspace, but the females will prefer to mate with this individual. Hence, the PL emissions play a key role in the evolution of damselflies. They can strongly help to enhance or modify the colors or colorations of the various colored-body portions, as biological pigments^13^,^14^,^15^ and multilayer structures^16^,^17^,^18^,^19^,^20^. The PL emissions from the cuticle of the various colored-body portions of the male damselfly *Ischnura senegalensis* range between 450 and 550 nm, and the maximum intensities reach ~1.0 × 10^4^ counts per second. The PL and micro-PL confocal images verify that the PL emissions can strongly influence the surface colors of the cuticle, and show why the damselfly *Ischnura senegalensis* is called a bluetail.

## Optical and PL Image of a Male Damselfly ***Ischnura Senegalensis*** (Rambur, 1842)

**Figure 1:**
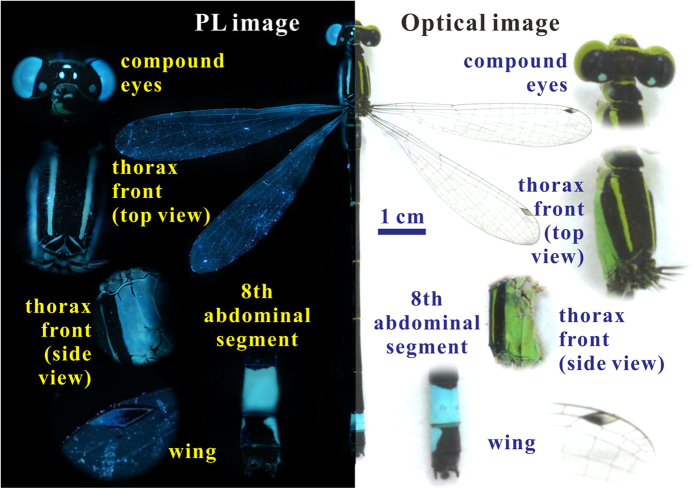
Merged image of a male damselfly *Ischnura senegalensis* (Rambur, 1842). The PL and optical images in the left and right-hand panels show the male damselfly and its various colored-body portions. Only the PL image shows the PL emissions from the colored-body portions that perform as special color signals. The insets display high-magnification PL and optical images of the compound eyes, thorax front, eighth abdominal segment, and wings, respectively. In the high-magnification optical images, the compound eyes, thorax front, and wings appear light green, but the eighth abdominal segment is light blue. In the high-magnification PL images, all the colors of these body parts, compound eyes, thorax front, eighth abdominal segment, and wings, look light blue, verifying that the PL emissions have a strong effect on the colors and coloration.

The merged image of the male damselfly *Ischnura senegalensis*, shown in Fig. 1, consists of a PL image (left panel) and an optical image (right panel). Both the PL and optical images demonstrate the four various colored-body portions of the male damselfly, but only the PL image can further show the PL emissions from the four various colored-body portions. Obviously, the four body parts, such as the compound eyes, thorax front, eighth abdominal segment and wings, are more colorful than the others, and it is supposed that these perform roles as special signals for the damselfly *Ischnura senegalensis*. In fact, a comparison of the PL and optical images shows that the colors of the colored-body portions look different in the PL image and the optical image. In other words the PL emissions modify the colors or coloration of the colored-body portions. PL and optical images of the compound eyes, thorax front, eighth abdominal segment, and wings are displayed in the insets to Fig. 1. In the optical images, the compound eyes and thorax front are light green, but the eighth abdominal segment and wings are light blue. However, in the corresponding PL images, the compound eyes, thorax front, eighth abdominal segment, and wings all appear light blue, verifying that the PL emissions have a huge effect on the cuticle colors and coloration. Specifically, the PL emissions enhance the visual contrast and modify the hues, so that the eighth abdominal segment can be easily recognized in the wild. Note that it can be clearly that the PL emissions are unevenly distributed along the wings in Fig. 1. This is because the wings, basically transparent thin films, reflect light at some incident angles, but allow most to easily pass through them. Thus, the PL emissions of the wings came from the veins of the wings.

## Pl Emissions of Various Colored-Body Portions

**Figure 2:**
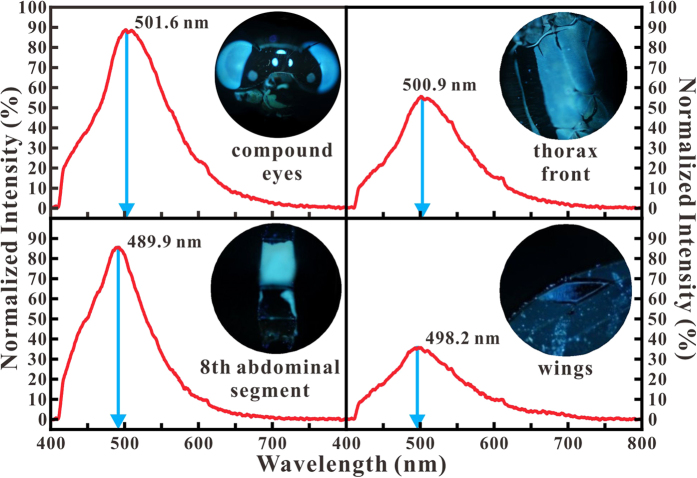
PL spectra for colored-body portions. The main PL intensity peaks of the compound eyes, thorax front, eighth abdominal segment, and wings are located at 501.6, 500.9, 489.9, and 498.2 nm, respectively. The PL emissions of the eighth abdominal segment make them appear as light blue in color, but the compound eyes and thorax front appear to be greenish or cyan in color. This is the reason why the damselfly *Ischnura senegalensis* is also called the bluetail.

The linear graphs in Fig. 2 show the four PL spectra for colored-body portions of the compound eyes, thorax front, eighth abdominal segment, and wings. The main PL intensity peaks for the compound eyes, thorax front, eighth abdominal segment, and wings are located at 501.6, 500.9, 489.9, and 498.2 nm, respectively. Only the wavelength of the main PL intensity peak of the eighth abdominal segment is smaller than 490 nm; those of the other colored-body portions are around 500 nm. To the human eyes, light at wavelengths around 490 and 500 nm looks blue and cyan. As known, blue is one of three primary colors, and cyan is a greenish color. The wavelengths of blue and cyan range from 450–495 and 490–520 nm, respectively. Hence, the PL emissions mean that the eighth abdominal segment appears to be light blue in color, while the compound eyes and thorax front appear greenish or cyan. That coloration is the reason why the damselfly *Ischnura senegalensis* is also called a bluetail. In the PL images, it can be seen that the eyes and eighth abdominal segment have a relatively strong PL intensity in contrast to the thorax front and wings, so they look brighter. They behave as color signals or cues. When two male damselflies meet each other, they can read the color signals to demonstrate the strength of their potential rival and whether they can be challenged or not. Also, when female damselflies meet the males, they can read the color signals to know whether that male is healthy and thus suitable for mating or not. In summary, the PL emissions play an important role as color signals that ensure the survival of the damselfly *Ischnura senegalensis*.

## Color Space Chromaticity Diagram of Various Colored-Body Portions

**Figure 3:**
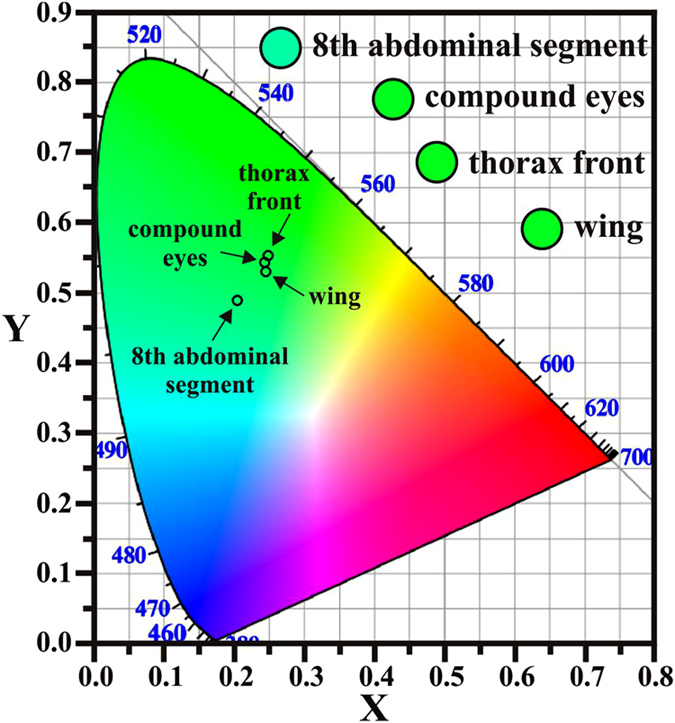
Color space chromaticity diagram for colored-body portions. The colors of the PL emissions from the colored-body portions of the compound eyes, thorax front, eighth abdominal segment, and wings, respectively, are shown. Obviously, the three colored-body portions of the compound eyes, thorax front, and wings appear a very similar green, while the eighth abdominal segment appears to be cyan-blue in both optical and PL images. The PL emissions strongly dominate to produce the perceived hue.

The colors of the PL emissions from the colored-body portions of the compound eyes, thorax front, eighth abdominal segment, and wings, are shown in the color space chromaticity diagram in Fig. 3. Three of the colored-body portions, the compound eyes, thorax front, and wings, appear to be a very similar green, although the intensities and band positions of the three PL spectra are different. The green color is what we would see in a bright room or space. It is known that biological pigments^13^,^14^,^15^ and multilayer structures^16^,^17^,^18^,^19^,^20^ can modify the colors or hues of insect surfaces. The color space chromaticity results indicate that the biological pigments and multilayer structures mainly contribute to the coloration of the compound eyes, thorax front, and wings. However, the eighth abdominal segment appears to be cyan-blue in the color space chromaticity results as well as in both optical and PL images, implying that it is not only the biological pigments and multilayer structures which act to modify the colors or hues of the colored-body portions. The coloration of the eighth abdominal segment is a good example of how the PL emissions strongly dominate to produce the perceived hue. Since the eighth abdominal segment has the same biological pigmentation and similar multilayer structures to those of the compound eyes, thorax front, and wings, the four colored-body portions should have the same coloration. However, the color of the eighth abdominal segment is different from that of the compound eyes, thorax front, and wings. Hence, it can be seen that PL emissions with wavelengths smaller than 490 nm may modify the colors or hues of the eighth abdominal segment from green to cyan-blue. The coloration of the colored-body portion is influenced not only by the biological pigment, or multilayer structures, but also by the PL emissions.

## Micro-PL Confocal and TEM Images of the Eighth Abdominal Segment

**Figure 4:**
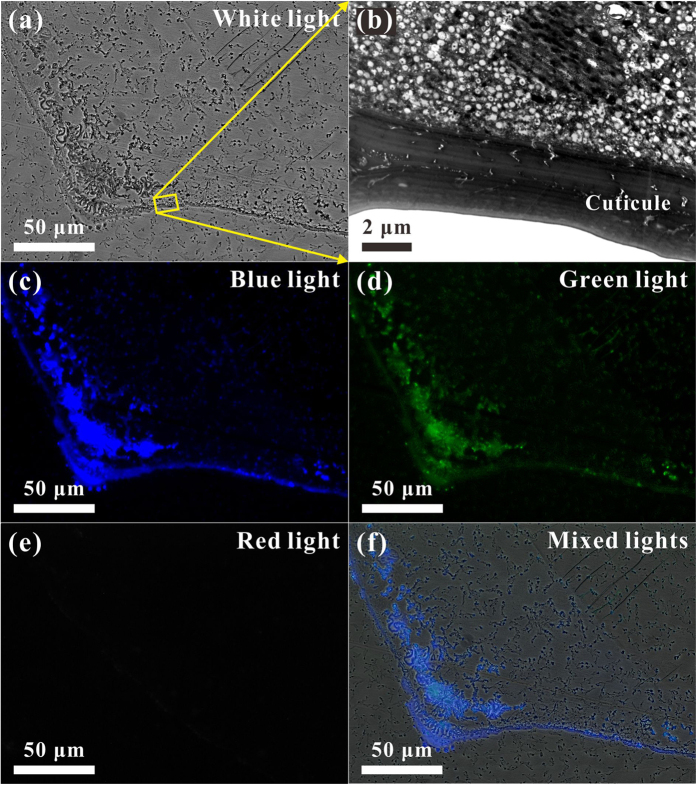
Micro-PL confocal and TEM images of the ultrathin histological sections of the eighth abdominal segment. (**a**) The micro-PL confocal image taken at white light displays the ultrathin histological section of the eighth abdominal segment. (**b**) The TEM image shows a high-magnification portion of the cuticle and internal structure, which is corresponding to the region highlighted by the yellow rectangular box in (**a**). (**c–e**) The micro-PL confocal images display the ultrathin histological section of the eighth abdominal segment taken at blue, green, and red lights, respectively. Obviously, only blue and green PL lights emit from the cuticle. (**f**) The micro-PL confocal image taken with the mixed lights (blue, green, and red lights) illustrates the eighth abdominal segment to be light blue in color.

To understand the impact of cuticle photoluminescence on the color morphism of a male damselfly *Ischnura senegalensis*, the ultrathin histological sections of the various specific body portions of the damselfly *Ischnura senegalensis* were examined by confocal fluorescence microscopy and TEM. Figure 4 shows micro-PL confocal and TEM images of the ultrathin histological sections of the eighth abdominal segment. In Fig. 4(a), the micro-PL confocal image taken at white light displays the ultrathin histological section of the eighth abdominal segment. It is easy to distinguish the cuticle and internal structure of the damselfly *Ischnura senegalensis*. The TEM image in Fig. 4(b) shows a high-magnification portion of the cuticle and internal structure. Note that the high-magnification portion corresponds to the region highlighted by the yellow rectangular box in Fig. 4(a). The cuticle is a multilayer structure, which can influence the surface colors^16^,^17^,^18^,^19^,^20^. The micro-PL confocal images in Fig. 4(c–e) display the ultrathin histological section of the eighth abdominal segment taken at blue, green, and red lights, respectively. Obviously, only blue and green PL lights emit from the cuticle, so the micro-PL confocal image in Fig. 4(f) taken with the mixed lights (blue, green, and red lights) illustrates the eighth abdominal segment to be light blue in color. The micro-PL confocal results verify why the damselfly *Ischnura senegalensis* is called a bluetail, and confirm that the PL emissions can also strongly influence the surface colors of the cuticle, as discussed above.

## Micro-PL Confocal and TEM Images of the Sixth Abdominal Segment

**Figure 5:**
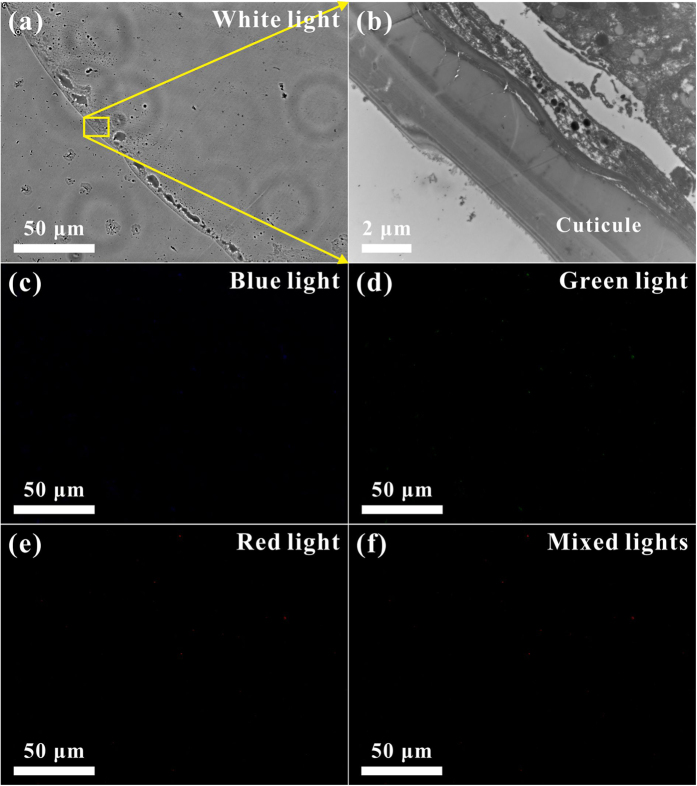
Micro-PL confocal and TEM images of the ultrathin histological sections of the sixth abdominal segment. (**a**) The micro-PL confocal image taken at white light displays the ultrathin histological section of the sixth abdominal segment. (**b**) The TEM image shows a high-magnification portion of the cuticle and internal structure, which is corresponding to the region highlighted by the yellow rectangular box in (**a**). (**c–f**) The micro-PL confocal images display the ultrathin histological section of the sixth abdominal segment taken at blue, green, red, and mixed lights, respectively. Apparently, no PL lights emit from the cuticle, so the cuticle color of the sixth abdominal segment looks black or khaki, not light blue.

The body portion of the thorax front has similar PL emissions from the cuticle, so the micro-PL confocal and TEM images of the ultrathin histological sections (as shown in Fig. S3, Supplemental Information) also confirm the PL emissions can strongly influence the surface colors of the cuticle. However, some body portions, such as the sixth and seventh abdominal segments, cannot emit any PL lights. Figure 5 shows micro-PL confocal and TEM images of the ultrathin histological sections of the sixth abdominal segment. In Fig. 5(a), the micro-PL confocal image taken at white light displays the ultrathin histological section of the sixth abdominal segment. Clearly, the cuticle and internal structure of the damselfly *Ischnura senegalensis* can be easily recognized. The TEM image in Fig. 5(b) shows a high-magnification portion of the cuticle and internal structure. Note that the high-magnification portion corresponds to the region highlighted by the yellow rectangular box in Fig. 5(a). The cuticle is also a multilayer structure, which can influence the surface colors^16^,^17^,^18^,^19^,^20^, as mentioned above. The micro-PL confocal images in Fig. 5(c–f) display the ultrathin histological section of the sixth abdominal segment taken at blue, green, red, and mixed lights, respectively. Apparently, no PL lights emit from the cuticle, so the cuticle color of the sixth abdominal segment looks black or khaki, not light blue. Notably, the cuticle color of the seventh abdominal segment is the same as that of the sixth abdominal segment. The micro-PL confocal and TEM images of the ultrathin histological sections are shown in Fig. S4 (Supplemental Information). No PL lights emit from the cuticle of the seventh abdominal segment. The no-PL results confirm again that the PL emissions can also strongly influence the surface colors of the cuticle, and verify that the cuticle PL does impact on the color morphism of a male damselfly *Ischnura senegalensis*.

## Summary

The Damselfly *Ischnura senegalensis* (Rambur, 1842) was first found to emit strong PL emissions from the various colored-body portions of the compound eyes, thorax front, wings, and eighth abdominal segment of the tail. There is an obvious difference in appearance of the colors of the colored-body portions in the PL image and those in the optical image, indicating that the PL emissions act as biological pigments and multilayer structures and can modify the colors or coloration of the colored-body portions. This PL phenomenon is strongly related to cuticle evolution in the damselflies. The ultrathin histological sections of the various specific body portions of the damselfly *Ischnura senegalensis* were examined by confocal fluorescence microscopy and TEM. Micro-PL confocal and TEM images of the ultrathin histological sections of the various specific body portions show that The cuticle PL does impact on the color morphism of a male damselfly *Ischnura senegalensis*, and it helps to reduce intrasexual and male harassment and to signal mature mating and conspecific identity.

## Methods

### Animals

**Figure 6:**
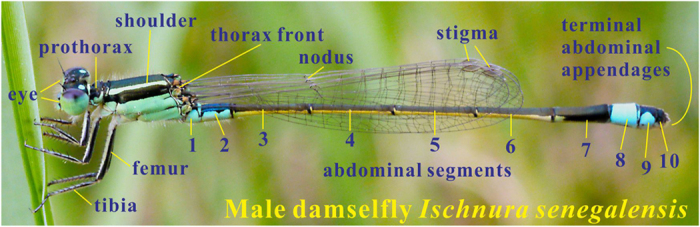
Photograph of a male damselfly *Ischnura senegalensis* (Rambur, 1842). This male damselfly *Ischnura senegalensis* was found and caught at the lakeside on the campus of National Dong Hwa University, Hualien, Taiwan, in June. The various specific body portions are indicated. The photograph was taken by the first author, Dr. Chin-Jung Chuang, at the lakeside on the campus National Dong Hwa University with a high-pixel camera (Panasonic Lumix DMC-GH1, 14 million total pixels).

A large number of species of damselflies and dragonflies inhabit the marshes along the lakeside of the campus of National Dong Hwa University, Hualien, Taiwan. *Ischnura senegalensis* (Rambur, 1842) is one of them, and can be easily seen and caught during the period from May to September. The male *Ischnura senegalensis* (Rambur, 1842) specimens were collected in 2015. The various specific body portions of captured damselflies, whose names are indicated in Fig. 6, can be directly observed by naked eyes or cameras.

### Anatomy and ultrastructural analysis

The male damselfly specimens were dehydrated through a series of acetone and ethanol solutions, and then immersed in propylene oxide for 10 minutes to remove residual acetone and ethanol. The dehydrated damselfly specimens were infiltrated and embedded in a resin, and then were cut into thin histological sections of only 80 nm thick with an ultramicrotome (Leica EM UC6) for various specific body portions of the male damselfly *Ischnura senegalensis* (Rambur, 1842). The ultrastructural analysis of the various specific body portions were carried using a transmission electron microscope (TEM, Hitachi H-7500). The ultrathin histological sections were loaded on 100-mesh copper grids in the TEM vacuum chamber, and then the cross-sectional structures of the ultrathin histological sections were observed at 80 kV accelerating voltages.

### PL spectromicroscopy

**Figure 7:**
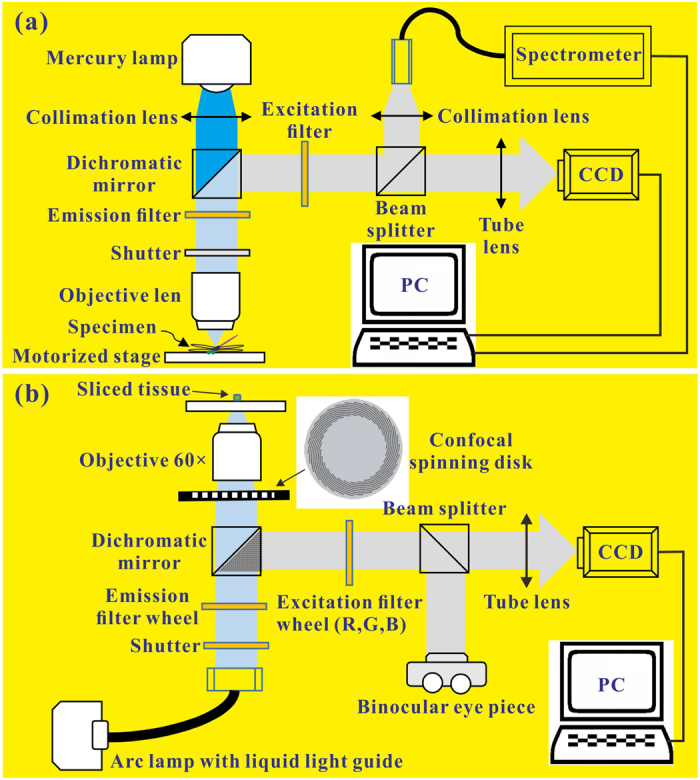
Setups of PL spectromicroscopy and confocal fluorescence microscopy. (**a**) The PL spectromicroscope mainly consists of an inverted-system optical microscope, a fiber optical spectrometer, a mercury arc lamp, and a CCD camera, and can provide PL images and spectra for the various specific body portions of the male damselfly *Ischnura senegalensis*. (**b**) The confocal fluorescence microscope mainly consists of an inverted-system optical microscope, a mercury/metal halide light source, a confocal spinning disk, and a high quantum efficiency CCD camera, and supply micro-PL confocal images for he ultrathin histological sections of the various specific body portions.

The various specific body portions of captured damselflies were directly observed by a PL spectromicroscope. The schematic diagram in Fig. 7(a) shows the setup of the PL spectromicroscope, which mainly consists of an inverted-system optical microscope (Olympus IX73), a fiber optical spectrometer (Ocean optics USB4000), a 100-Watt mercury arc lamp (Olympus U-RFL-T), and a charge-coupled-device (CCD) camera. The mercury arc lamp could provide light ranging from UV to infrared (IR). The high PL emissions of the colored-body portions were excited by the UV light, and then passed through a fluorescence filter cube consisting of an excitation filter (FF01-370/36-25, Semrock), an emission filter (FF02-409/LP-25, Semrock), and a dichroic beam splitter (Di02-R405-25 × 36, Semrock). Note that one second of exposure time was sufficient to obtain the PL emissions with a high signal to noise ratio, and avoid light damage. PL images and spectra were easily obtained by the optical microscope and fiber optical spectrometer, respectively. The compound eyes and eighth abdominal segments provided relatively strong PL emissions, unlike the other colored-body portions.

### Confocal fluorescence microscopy

The ultrathin histological sections of the various specific body portions were perceived by a confocal fluorescence microscope (CARV II™ Confocal Imager). The schematic diagram in Fig. 7(b) shows the setup of the confocal fluorescence microscope, which mainly consists of an inverted-system optical microscope (Olympus IX71S8F3) with a 60x objective, a 120-Watt mercury/metal halide light source, a confocal spinning disk, and a high quantum efficiency CCD camera (100 frames per second). The 120 W mercury/metal halide light source allows full-spectrum (360–700 nm) confocal imaging through filter sets matched to the excitation and emission requirements of PL (or fluorescent) specimens. The micro-PL confocal images of the ultrathin histological sections at the various specific body portions were taken under the bright field with and without blue, green and red filters, respectively. These taken micro-PL confocal images were saved and processed with VisiView^®^ Imaging Software.

## Additional Information

**How to cite this article**: Chuang, C.-J. *et al*. Impact of cuticle photoluminescence on the color morphism of a male damselfly *Ischnura senegalensis* (Rambur, 1842). *Sci. Rep.* **6**, 38051; doi: 10.1038/srep38051 (2016).

**Publisher's note:** Springer Nature remains neutral with regard to jurisdictional claims in published maps and institutional affiliations.

## Supplementary Material

Supplemental Information
