# Supplementary material for: Impact of cuticle photoluminescence on the color morphism of a male damselfly Ischnura senegalensis (Rambur, 1842)
Source: Sci Rep. 2016 Dec 14;6:38051. doi: 10.1038/srep38051 (PMC5155280; doi:10.1038/srep38051)
Supplement: Supplemental Information [file srep38051-s1.doc]

**Supplemental Information**

**Impact of cuticle photoluminescence on the color morphism of a male damselfly *Ischnura senegalensis* (Rambur, 1842)**

Chin-Jung Chuang^1^, Cheng-Der Liu,^2^ Ranjit A. Patil^3^, Chi-Chung Wu^1^, Yao-Chih Chang^1^, Chih-Wen Peng,^2^ Ting-Kwuan Chao^2^, Je-Wen Liou^4^, Yung Liou^5^, and Yuan-Ron Ma^3^,*

^1^Department of Opto-Electronic Engineering, National Dong Hwa University, Hualien, 97401, Taiwan.

^2^Institute of Medical Sciences, Tzu Chi University, Hualien 97004, Taiwan

^3^Department of Physics, National Dong Hwa University, Hualien, 97401, Taiwan.

^4^Department of Biochemistry, School of Medicine, Tzu Chi University, Hualien 97004, Taiwan

^5^Institute of Physics, Academia Sinica, Taipei, 11529, Taiwan.

*Corresponding author

Yuan-Ron Ma, Professor, Department of Physics, National Dong Hwa University, No. 1, Sec. 2, Da-Hsueh Rd., Shou-Feng, Hualien 97401, Taiwan.

[Tel:+886-3-8633706](tel:+886-3-8633706). Fax: +886-3-8633690. Email: [ronma@mail.ndhu.edu.tw](mailto:ronma@mail.ndhu.edu.tw)

Keywords: damselfly, *Ischnura senegalensis*, photoluminescence, coloration, confocal fluorescence.


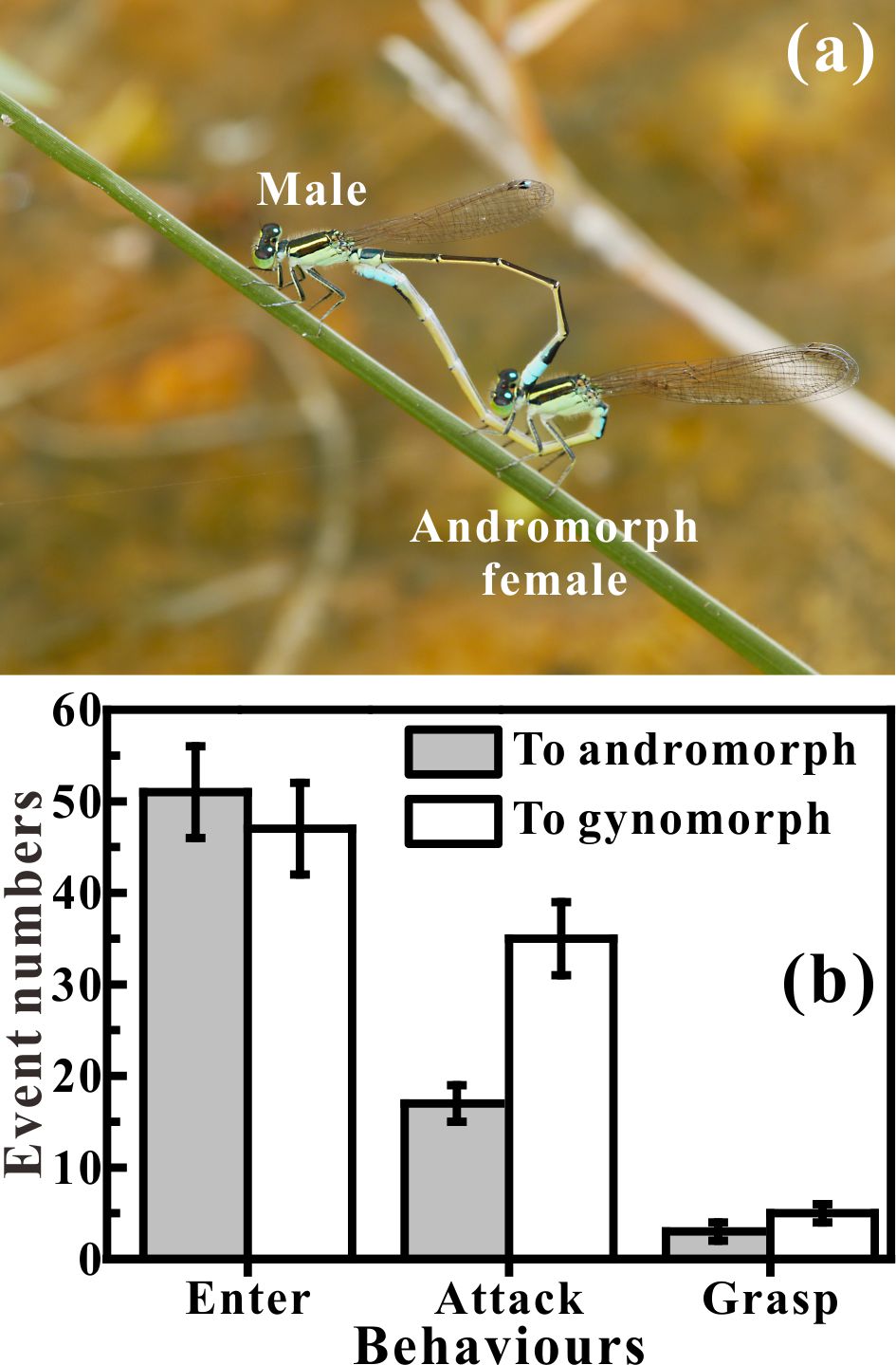


**Fig. S1 | Mating and harassment statistics of damselflies *Ischnura senegalensis* (Rambur, 1842).** (a) The female damselfly (lower) exhibits color dimorphism, namely andromorph and gynomorph. The andromorph acts to reduce intrasexual and male harassment and to achieve mature mating. The andromorphic colors from the colored-body portions provide good visual contrast for conspecific identity in the ambient light in the varying environmental background. The photograph was taken by the first author, Dr. Chin-Jung Chuang, at the lakeside on the campus of the National Dong Hwa University with a high-pixel camera (Panasonic Lumix DMC-GH1, 14 million total pixels). (b) The harassment events of enter, attack and grasp were counted for the male damselflies *Ischnura senegalensis* harassing the andromorph and gynomorph female damselflies in 6 hours from 9am to 15pm. There are 161 events in total; 50 and 48 enter events, 17 and 38 attack events, and 4 and 6 grasp events for andromorph and gynomorph, respectively. The results of the attack events verify that the andromorph helps to reduce intrasexual and male harassment and allows them to gain mature mating.


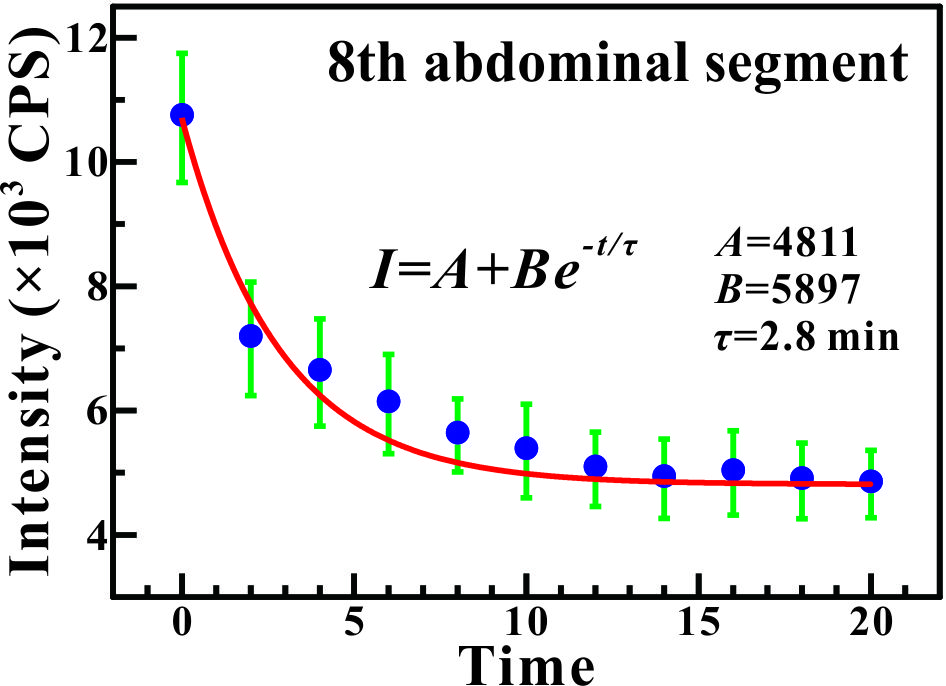


**Fig. S2 | PL intensity decay with time.** There is a dramatic decay in the PL intensity of the eighth abdominal segment in 20 min after death. The PL intensity decay can be curved-fitted using an exponential function of time. The exponential function is *I=A+Be^-t/τ^*, where *I* represents the PL intensity, *A* is the initial constant, and *B* and *τ* are the curve-fitted constants, respectively. Therefore, the intensity of the PL emission can be used to indicate the good health and strength of the damselfly.

**
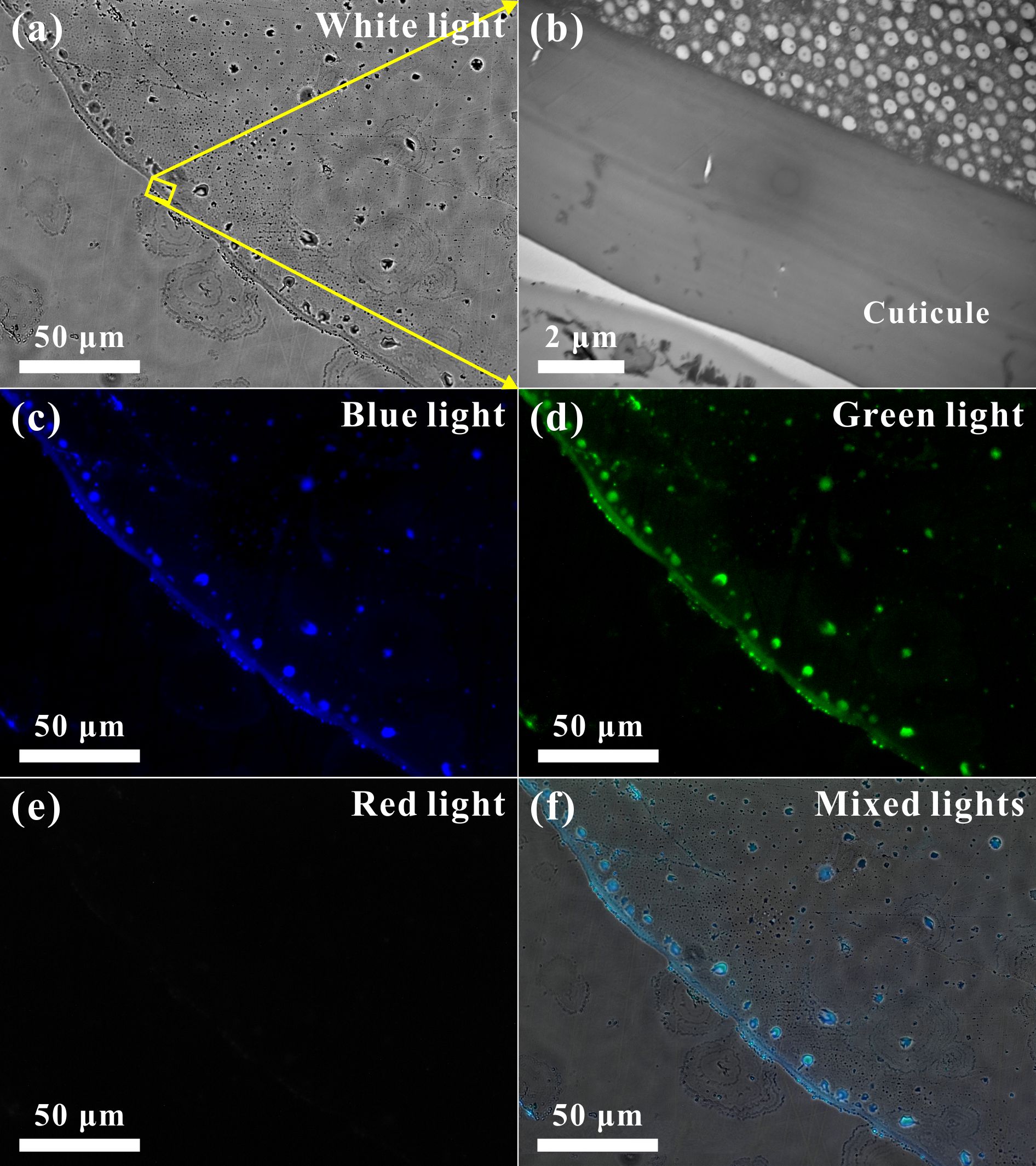
**

**Fig. S3 | Micro-PL confocal and TEM images of the ultrathin histological sections of the thorax front.** (a) The micro-PL confocal image taken at white light displays the ultrathin histological section of the thorax front. (b) The TEM image shows a high-magnification portion of the cuticle and internal structure, which is corresponding to the region highlighted by the yellow rectangular box in (a). (c-e) The micro-PL confocal images display the ultrathin histological section of the thorax front taken at blue, green, and red lights, respectively. Obviously, only blue and green PL lights emit from the cuticle. (f) The micro-PL confocal image taken with the mixed lights (blue, green, and red lights) illustrates the thorax front to be cyan in color.

**
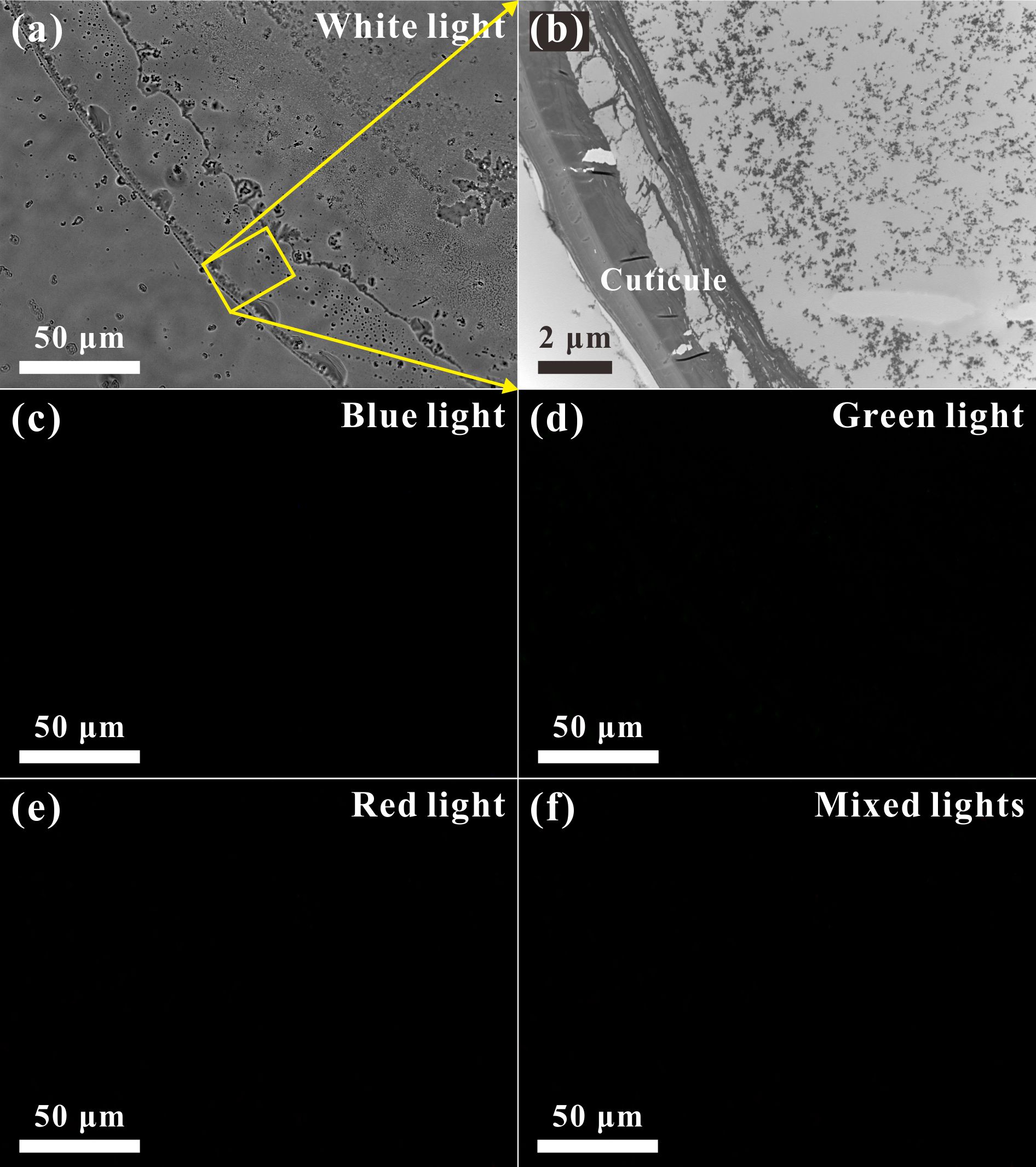
**

**Fig. S4. Micro-PL confocal and TEM images of the ultrathin histological sections of the seventh abdominal segment.** (a) The micro-PL confocal image taken at white light displays the ultrathin histological section of the seventh abdominal segment. (b) The TEM image shows a high-magnification portion of the cuticle and internal structure, which is corresponding to the region highlighted by the yellow rectangular box in (a). (c-f) The micro-PL confocal images display the ultrathin histological section of the seventh abdominal segment taken at blue, green, red, and mixed lights, respectively. Apparently, no PL lights emit from the cuticle, so the cuticle color of the seventh abdominal segment looks black or khaki, not light blue.
